# Transient Third-Degree Atrioventricular Block in a Dog with Addisonian Crisis

**DOI:** 10.3390/vetsci12010063

**Published:** 2025-01-16

**Authors:** Paula Maria Pașca, Gheorghe Solcan, Andrei Blageanu, Caroline Maria Lăcătuș, Petru Cosmin Peștean, Constantin Adrian Stancu, Andrei Radu Baisan

**Affiliations:** 1 Department of Clinics, Faculty of Veterinary Medicine, “Ion Ionescu de la Brad” Iasi University of Life Sciences, 700490 Iasi, Romania; paula.pasca@iuls.ro (P.M.P.); andrei.blageanu@gmail.com (A.B.); andrei.baisan@iuls.ro (A.R.B.); 2 Faculty of Veterinary Medicine, University of Agricultural Sciences and Veterinary Medicine, 400372 Cluj-Napoca, Romania; carolinelacatus@yahoo.com (C.M.L.); cosmin.pestean@usamvcluj.ro (P.C.P.); 3 Department of Pathology, University of Life Sciences “King Mihai I”, 300645 Timișoara, Romania; adrianstancu@usvt.ro

**Keywords:** Addison’s disease, adrenal glands, canine, complete atrioventricular block, hyperkalemia

## Abstract

A 3-year-old spayed male mixed-breed Labrador presented to the Emergency and Critical Care Unit with lethargy, loss of appetite, vomiting, a recent history of presyncopal episodes, and severe exercise intolerance. On admission, the patient had bradycardia, low blood pressure, and mild abdominal pain. Serum biochemistry information revealed severe hyperkalemia, hyponatremia, hypoglycemia, and mildly increased liver and kidney parameters. Upon taking these findings into consideration, hypoadrenocorticism was suspected, and a basal cortisol level was determined; it was below 1.0 µg/dL. Upon examination with ultrasound, we observed that the size of the adrenal glands was considerably reduced. Due to the bradycardia, a complete cardiologic examination consisting of 5 min of six-lead electrocardiography and echocardiography (ECG) was performed. Six-lead ECG revealed a complete atrio-ventricular block with an atrial rate of 140 bpm and a ventricular rate of 60 bpm. The PQ interval was variable, without any evidence of atrio-ventricular conduction. Treatment for hyperkalemia was initiated immediately after admission, followed by dexamethasone sodium phosphate administration, which led to an improvement in the patient’s general condition, and the normalization of the ECG readings, after 24 h of therapy. Afterward, prednisolone (0.5 mg/kg/24 h p.o.) and desoxycorticosterone pivalate (2.2 mg/kg s.c.) were administered. This case shows that a third-degree atrioventricular block might be a reversible problem in dogs with hypoadrecorticism that can be managed with specific treatment for this disorder, without requiring pacemaker implantation.

## Simple Summary

Hypoadrenocorticism, or Addison’s disease, is a rare endocrine disorder especially prevalent in young to middle-aged dogs. The clinical signs are inconclusive: lethargy, loss of appetite, vomiting, exercise intolerance, bradycardia, low blood pressure, abdominal pain, etc. Numerous electrolyte abnormalities may be seen in cases of hypoadrenocorticism, such as hyponatremia, hyperkalemia, hypochloremia, hypercalcemia, and metabolic acidosis. Hyperkalemia induces well-known electrocardiographic changes. We present an uncommon case of a dog suspected of having hypoadrenocorticism and hyperkalemia associated with a reversible complete atrio-ventricular block with narrow QRS complexes. In this case, the conversion occurred within a timeframe of 24 h, once the serum potassium levels normalized. This strongly suggests that the complete atrio-ventricular block was induced by hyperkalemia.

## 1. Introduction

Hypoadrenocorticism (HA), or Addison’s disease, is a rare endocrine disorder especially prevalent in young to middle-aged dogs, with a median age of diagnosis of 3 to 4 years. It is caused by a dysfunction of the adrenal cortex, which fails to produce the necessary quantities of glucocorticoids and mineralocorticoids, essential for survival. Clinical signs are usually nonspecific and range from weakness, anorexia, vomiting, diarrhea, and shaking, which are mostly attributed to the lack of cortisol, but approximately 30% of dogs with HA present with acute Addisonian crisis, with signs of hypovolemic shock, which include bradycardia or tachycardia, collapse, hypothermia, weak pulses, and a prolonged capillary refill time [1,2,3].

Numerous electrolyte abnormalities may be seen in HA, such as hyponatremia, hyperkalemia, hypochloremia, hypercalcemia, and metabolic acidosis. Hyperkalemia and hyponatremia are encountered in over 80% of cases of dogs with HA. The Na:K ratio is a useful parameter for helping medical personnel test for HA and also monitor the response to therapy [3,4,5].

Hyperkalemia induces well-known electrocardiographic (ECG) changes. Increased potassium levels cause the resting membrane potential to shift towards less-negative levels and decrease the slope of phase 0 [6]. Generally, ECG changes during hyperkalemia are visible when the potassium levels increase above 6–6.5 mmol/L and include tall and peaked T-waves, a shortened QT interval, and a prolongation and decrease in the amplitude of the P wave. In more severe conditions, hyperkalemia induces bradycardia, a sino-ventricular rhythm, and a widening of the QRS complex [6].

## 2. Case Description

A 3-year-old, 27 kg mixed-breed spayed male Labrador presented to the Emergency and Critical Care Unit of the Faculty of Veterinary Medicine, University of Life Sciences, Iasi, with lethargy, loss of appetite, vomiting, a recent history of presyncopal episodes, and severe exercise intolerance. On admission, a physical examination revealed a normal body condition score (4/9), a normal respiratory frequency (36 resp./min) with a normal respiratory pattern, and subicteric mucous membranes. Palpation revealed mild abdominal pain. Upon auscultation, bradycardia (52 beats/min) was detected, with a regular rhythm and no murmurs, and the results of lung auscultation were unremarkable. The subject’s rectal temperature was 38 °C, and its blood pressure measured using an oscyllometric (Vet HDO Blood Pressure Monitor, S+B medVet GmbH, Babenhausen, Germany) method was 105/62 mmHg (S/D).

The patient was vaccinated and dewormed and had undergone preventive external parasite treatment two weeks earlier. It had not received any other treatments or supplements and had no previously known pathologies prior to admission to the hospital. Blood samples were collected from the jugular vein to perform a complete blood count (CBC) and serum biochemistry. The complete blood count, performed using Abaxis VetScan HM5 (Zoetis Services, Parsippany, NJ, USA), showed polycythemia with a red blood cell count of 11.52 × 10^6^/mcL [reference interval (RI) 5.5–8.5 × 10^6^/mcL], a hematocrit of 74.54% (RI 37–55%), and a hemoglobin level of 28.1 mg/dL (RI 12–18 mg/dL).

The serum biochemistry analysis, performed using Abaxis VetScan V2 (Zoetis Services, Parsipanny, NJ, USA)-VetScan Comprehensive Diagnostic Profile, indicated mildly 3 elevated liver enzyme levels, with an aspartate aminotransferase level of 111 U/L (RI 10–118 U/L), a phosphorus level of 6.24 mg/dL (RI 2.9–6.6 mg/dL), a creatinine level of 1.61 mg/dL (RI 0.3–1.4 mg/dL), a hypoglycemia value of 65 mg/dL (RI 60–110 mg/dL), a hyperkalemia value of 7.9 mmol/L (RI 3.7–5.8 mmol/L), and a hyponatremia value of 123.1 mmol/L (RI 138–160 mmol/L). The Na:K ratio was 15.5. Its troponin level, determined using a point-of-care immunofluorescence analyzer (Vcheck V200, Bionote, Hwaseong-si, Republic of Korea), was 0.43 ng/mL, while the normal limit is considered to be a value below 0.03 ng/mL.

A test （SNAP 4Dx Plus Test, IDEXX, Westbrook, ME, USA) for vectorial-borne diseases (heartworm, Lyme disease, Anaplasma, and Ehrlichia) and a blood smear examination were performed, both of which came back negative.

**Figure 1:**
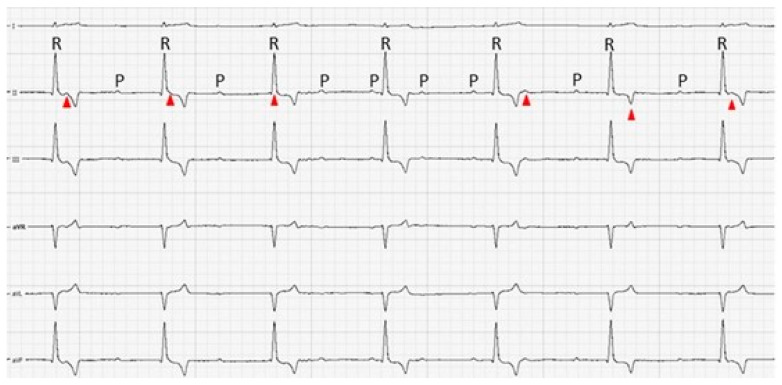
Six-lead electrocardiography recording from a 3-year-old, 27 kg mixed-breed male Labrador indicating complete atrio-ventricular dissociation with narrow QRS complexes and a normal mean electrical axis in the frontal plane. The red arrowheads represent the P-waves hidden in the QRS, ST segment, or T-wave; note that the PQ interval is variable, suggesting complete dissociation between the sinus and the ventricular rhythm; P—p-wave; R—QRS complex; calibration: 50 mm/sec and 10 mm/mV.

Due to the bradycardia, a complete cardiologic examination consisting of 5 min six-lead electrocardiography (Polyspectrum 8E/8V, Ivanovo, Russia) and echocardiography (Logiq V5 Expert, General Electric, Wuxi, China) was performed, with the patient being placed in the right lateral recumbent position. Six-lead ECG revealed a complete atrio-ventricular block with an atrial rate of 140 bpm and a ventricular rate of 60 bpm (Figure 1). The PQ interval was variable, and there was no evidence of atrio-ventricular conduction. The P-waves were small, with an amplitude of 0.04 mV. The QRS complexes had a similar morphology over the entire recording period, with positive polarity at the inferior leads (II, III, and aVF) and negative polarity in aVR, a mean electrical axis of 83^0^, an amplitude of 1 mv, and a duration of 75 msec, consistent with an idioventricular rhythm with a proximal localization, probably within the proximal area of the His bundle. There were no other types of arrhythmias detected in the 5 min ECG recording.

Echocardiography revealed normal dimensions of the left side of the heart. The right side of the heart was subjectively assessed and showed unremarkable changes. The left-atrium/aorta (LA/Ao) ratio in the right parasternal short axis view at the heart base one frame after the closure of the aortic valves was determined to be 1.21 (normal < 1.6) [7]. The left-ventricular internal diameter normalized to bodyweight [8] in diastole (LVIDd_n) was 1.31 (normal 1.27–1.85), and that in systole (LVIDs_n) was 0.89 (normal 0.71–1.26). The LV SF% was 35.8%. The pulmonary and aortic flows were laminar, with maximum velocities of 1.03 m/s and 1.14, respectively. The E-wave had a maximum velocity of 0.6 m/s. There was no evidence of mitral or tricuspid valve insufficiency. Considering the findings of the clinical examination and the results of the blood work, Addison’s disease was suspected. Since ACTH (corticotrophin) analogues were not available in Romania at the time of this study, a basal cortisol level was determined (Vcheck V200, Bionote, Hwaseong-si, Republic of Korea), which was below 1 µg/dL, a value suggestive of hypoadrenocorticism [9].

Treatment was initiated with aggressive intravenous fluid therapy with lactated Ringer’s solution (Lactated Ringer’s solution, Braun, Melsungen, Germany) at a rate of 18 mL/kg/h and management of hyperkalemia [10]. In the first phase of the treatment, calcium gluconate (Calcium Gluconate, 94 mg/mL, Braun, Melsungen, Germany) was administered at a dose of 0.5 mL/kg over 20 min under ECG monitoring. This treatment was followed by dextrose administration (Glucosio Baxter S.P.A., 50%, Deerfield, IL, USA). In this procedure, 50% dextrose was added to the balanced crystalloid to make a 2.5% solution of dextrose in order to maintain normoglycemia [1]. Afterwards, dexamethasone sodium phosphate (Dexametazone 4 mg/mL, Rompharm, Bucharest, Romania) was administered intravenously at a dose of 0.25 mg/kg/24 h [1].

**Figure 2:**
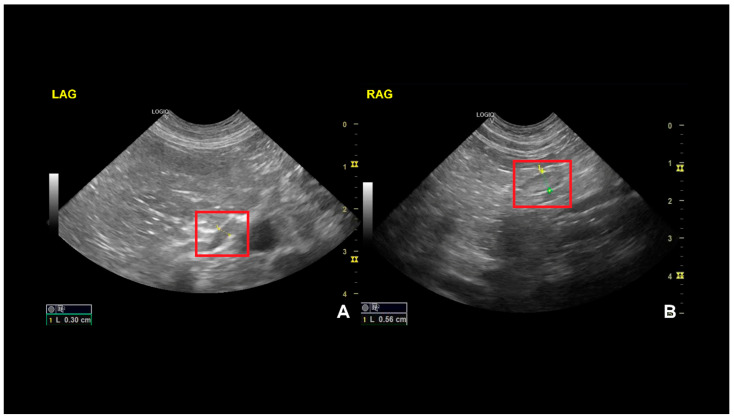
Adrenal gland ultrasound images of a dog, showing the longitudinal axis. On the left (**A**) is the left adrenal gland (LAG), with a diameter of 3 mm, corresponding to an ultrasound change consistent with HA; one the right (**B**) is the right adrenal gland (RAG), with a normal diameter of 5.6 mm.

An abdominal ultrasound was performed using a General Electric LOGIQ V5 Expert Ultrasound Machine (GE Medical Systems, Wuxi, China) equipped with a 7.5–10 MHz microconvex transducer and a 7–13 MHz linear transducer. The examination was conducted in a counter-clockwise direction, starting with the urinary bladder. Abdominal ultrasonography was performed according to the American College of Veterinary Radiology and European College of Veterinary Diagnostic Imaging consensus statement for the standardization of abdominal ultrasound examinations [11]. Except for the thickness of the left adrenal gland, there were no other remarkable findings. The left adrenal gland was visualized according to the following anatomic landmarks: medial and cranial to the apical pole of the left kidney and ventro–lateral to the aorta between the cranial mesenteric and the left renal arteries [12]. Color Doppler was used to differentiate the organ in question from the adjacent vasculature and find the phrenicoabdominal vein. The left adrenal gland had a normal shape and echogenicity, with a diameter of 3 mm on the longitudinal axis at the caudal pole (normal values: 5.3–6.3 mm) (Figure 2) [13]. The right adrenal gland was visualized according to the following anatomic landmarks: craniomedial to the renal hilus, lateral or dorsolateral to the caudal vena cava, and cranial to the right renal vein [14]. The right adrenal was normal-shaped and exhibited echogenicity, with a diameter of 0.56 cm on the longitudinal axis at the caudal pole (normal values: 5.9–6.9 cm) [15].

**Figure 3:**
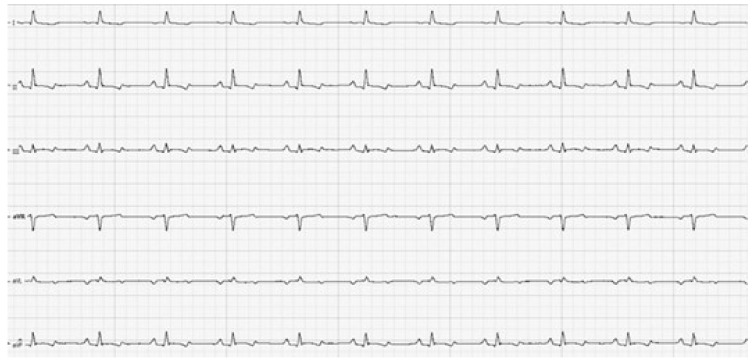
Six-lead electrocardiography results for the same dog after serum potassium concentration normalization: note the presence of a sinus rhythm with a mean heart rate of 110 bpm and constant PQ intervals with a duration of 100 msec; calibration: 50 mm/sec and 10 mm/mV.

There were two additional potassium determinations performed in the first 24 h after admission to the Hospital: they showed a continuous decrease in the potassium level, with a value of 7.2 mmol/L after almost 2 h and 6.4 mmol/L after 4 h. Twenty-four hours later, the ECG was repeated, revealing a sinus rhythm with normal atrio-ventricular conduction, with a mean HR over 5 min of 110 bpm (Figure 3). The P-wave was 0.18 mV in amplitude and 48 msec in duration. The PQ interval was 100 msec. The QRS complex was 0.74 mV in amplitude and 58 msec in length. The mean electrical axis was 51^0^. The subject’s potassium level at this time was 4.8 mmol/L.

Treatment was continued with prednisolone (0.5 mg/kg/24 h p.o.) (Prednicortone 20 mg tb, Dechra, Skipton, UK) and desoxycorticosterone pivalate (Zycortal 25 mg/mL, Dechra, Skipton, UK) (2.2 mg/kg s.c.), which led to the improvement of the patient’s general condition. The patient was discharged 72 h after admission and prescribed daily oral prednisolone administration. The dog came back for a check-up and routine blood monitoring after two weeks, and it was observed that its potassium levels were within normal limits (4.27 mmol/L), with a Na:K ratio of 33. The dog comes regularly to the Hospital for Zycortal injections, and every 3 to 4 months, blood tests are performed. Within 24 months after the diagnosis, the patient had normal potassium levels and no ECG abnormalities.

## 3. Discussion

The present case describes a severe arrhythmia in the context of an Addison crisis in a dog. Potassium plays an important role in cardiac depolarization and repolarization [6]. Hyperkalemia is associated with electrocardiographic changes depending on the K^+^ serum concentration, ranging from peaked and tall T-waves to prolongation and a decreased amplitude of the P-wave, a sino-ventricular rhythm, severe bradycardia, and ventricular asystole [6]. A third-degree atrio-ventricular block is an uncommon electrocardiographic arrhythmia associated with hyperkalemia in both humans [14,16] and dogs. There have only been four reported cases of a third-degree AV block in dogs with hyperkalemia secondary to hypoadrenocorticism [17,18,19,20]. In two of these cases, the arrhythmia did not resolve after hyperkalemia was managed [17,18]; however, in one case, the dog switched to a sinus rhythm after its K^+^ levels returned to normal. In this particular case, the dog also partially responded to atropine, suggesting there was also a vagal influence [20]. In the present case, an atropine test was not performed; therefore, it remains unknown whether a vagal component was associated with the bradyarrhythmia observed.

In the most recent published case, the arrhythmia resolved after treatment was administered and the potassium levels returned to normal limits. Also, the atropine response test, which is used to help differentiate intrinsic cardiac nodal disease from a vagally mediated bradyarrhythmia, did not reveal a change in heart rate [20].

Interestingly, as in our report, both cases with a complete atrioventricular block associated with hyperkalemia that converted to a sinus rhythm after reaching normal potassium levels showed a narrow QRS complex, suggesting that ventricular depolarization occurs in the distal part of the atrio-ventricular node or within the proximal part of the His bundle [19,20].

There is a wide range of normal sizes for the adrenal gland in dogs depending on the breed. However, studies show that adrenal glands below 0.32 cm combined with clinical signs suggestive of primary hypoadrenocorticism are strongly suggestive of disease [21,22].

In the present case, the diagnosis of Addison’s disease was difficult to make due to the unavailability of corticotrophin analogues, even for human medicine. Therefore, the diagnosis was suspected, and therapy was initiated without the gold-standard ACTH stimulation test [23].

Among the landmarks that helped orient the diagnosis was the Na:K ratio of 15.5 upon presentation, since, as demonstrated before by Adler et al., a ratio of 24 or less makes the likelihood of hypoadrenocorticism very high [24]. A second highlight was the basal cortisol value, which was below 1 µg/dL. The research conducted by Lennon et al. showed that basal cortisol concentrations of ≤1 μg/dL had a sensitivity of 100% and a specificity of 98.2% for detecting dogs with hypoadrenocorticism [9]. On the other hand, Bovens et al. recorded a sensitivity of 85.7% for basal serum cortisol concentration ≤ 1 µg/dL and a specificity of 91.8% for detecting hypoadrenocorticism [25], which underlines that a definitive diagnosis of hypoadrenocorticism should only be made after an ACTH stimulation test. Also, the ultrasound evaluation of the adrenal gland revealed a remarkably reduced size compared to values reported in the literature for healthy dogs in the same weight category [13,26].

Lastly, the therapeutic response along with potassium level normalization and sinus rhythm conversion, in the absence of any cardiac structural changes, were highly suggestive of hypoadrenocorticism. The patient has now been monitored for two years and has not shown any signs of relapse. Its Na:K was 32 on the last determination, and it has gained over 5 kg in weight. It is worth mentioning that the patient never exhibited any of the adverse effects of desoxycorticosterone pivalate observed when given to healthy dogs, such as polyuria, polydipsia, decreases in serum potassium and urea concentrations, transient increases in serum sodium concentrations, and significant decreases in body weight [27].

## 4. Conclusions

We have presented an uncommon case of a dog suspected of having hypoadrenocorticism and hyperkalemia associated with a reversible complete atrio-ventricular block with narrow QRS complexes. In this case, the conversion occurred within a timeframe of 24 h, once the serum potassium levels normalized. This strongly suggests that the complete atrio-ventricular block was induced by hyperkalemia.

## Data Availability

The data presented in this study are available in the manuscript.
